# Shelf Life and Simulated Gastrointestinal Tract Survival of Selected Commercial Probiotics During a Simulated Round-Trip Journey to Mars

**DOI:** 10.3389/fmicb.2021.748950

**Published:** 2021-10-07

**Authors:** Patricia Fajardo-Cavazos, Wayne L. Nicholson

**Affiliations:** Space Life Sciences Laboratory, Department of Microbiology and Cell Science, Exploration Park at Kennedy Space Center, University of Florida, Merritt Island, FL, United States

**Keywords:** *Bacillus subtilis*, *Bifidobacterium longum*, *Lactobacillus acidophilus*, probiotic, simulated Galactic Cosmic Radiation, simulated Solar Particle Event

## Abstract

To enhance the gastrointestinal health of astronauts, probiotic microorganisms are being considered for inclusion on long-duration human missions to the Moon and Mars. Here we tested three commercial probiotics—*Bifidobacterium longum* strain BB536, *Lactobacillus acidophilus* strain DDS-1, and spores of *Bacillus subtilis* strain HU58—for their survival to some of the conditions expected to be encountered during a 3-year, round trip voyage to Mars. All probiotics were supplied as freeze-dried cells in capsules at a titer of >10^9^ colony forming units per capsule. Parameters tested were survival to: (i) long-term storage at ambient conditions, (ii) simulated Galactic Cosmic Radiation and Solar Particle Event radiation provided by the NASA Space Radiation Laboratory, (iii) exposure to simulated gastric fluid, and (iv) exposure to simulated intestinal fluid. We found that radiation exposure produced minimal effects on the probiotic strains. However, we found that that the shelf-lives of the three strains, and their survival during passage through simulations of the upper GI tract, differed dramatically. We observed that only spores of *B. subtilis* were capable of surviving all conditions and maintaining a titer of >10^9^ spores per capsule. The results indicate that probiotics consisting of bacterial spores could be a viable option for long-duration human space travel.

## Introduction

NASA’s Human Research Program (HRP^1^) is currently planning for long-term human missions to destinations including the Moon and Mars. As described in the NASA HRP Requirements Document (Anonymous, 2015), for purposes of planning long-duration human missions into deep space, Design Reference Mission (DRM) categories specify the basic parameters of a mission such as its destination, space environment, gravity level(s), and duration. Each DRM scenario dictates essentially all vital parameters of a mission. For example, a typical 3-year DRM to Mars would entail exposure to microgravity and deep-space ionizing radiation for a total of 12–18 months on the outbound and return trips, and exposure to Mars gravity (0.376 × *g*) and increased ionizing radiation for an 18-month stay on the Martian surface.

Crucial to mission success is the safe delivery to Mars and return to Earth of a healthy astronaut crew capable of optimal performance of all assigned tasks. Long-duration excursions into the deep-space environment will expose astronauts to chronic stresses imposed by microgravity, ionizing radiation, and confinement. These aspects of spaceflight have been shown to alter astronaut physiology, affecting nearly every system including musculoskeletal, neurological, endocrine, cardiovascular, respiratory, excretory, cognitive, and immune systems (Demontis et al., 2017; Cekanaviciute et al., 2018; Crucian et al., 2018). Development of effective countermeasures to maintain astronaut health and performance under the extreme conditions of deep space exploration is an actively ongoing NASA endeavor (for details see NASA’s Human Research Roadmap (HRR;^2^).

In recent years it has been recognized that humans are actually metaorganisms, i.e., multispecies consortia consisting of the human host and its associated microbial inhabitants (the microbiome) (Bosch and McFall-Ngai, 2011). The human microbiome consists of hundreds of species found at various body sites—skin, oral, gastrointestinal, etc. (Cho and Blaser, 2012)—with the vast majority residing in the gastrointestinal (GI) tract (Sender et al., 2016). Recent years have seen a surge of research and a dramatic improvement of our understanding into how the host, its microbiome, and the environment interact to determine human health and disease on Earth, and these studies have recently been extended to the spaceflight environment. For example, two recent studies of astronauts in the ISS revealed evidence of both compositional and functional changes in the astronaut microbiome during long-duration spaceflight. In general, (i) astronauts’ skin microbiomes became more similar to the ambient ISS microbiome with time, and (ii) astronauts’ GI microbiomes tended to become more similar to one another, likely due in part to their common diet at the ISS (Garrett-Bakelman et al., 2019; Voorhies et al., 2019).

An open question for long-term human exploration centers around the possible use of probiotics to maintain a healthy astronaut GI tract. Probiotics are defined by the World Health Organization as “live microorganisms which when administered in adequate amounts confer a health benefit on the host” (Sanders, 2003). On Earth, probiotics have been shown to interact with the host and its GI microbiome to improve the immune response, protect against pathogens, and improve gut barrier function (Suez et al., 2019; Cunningham et al., 2021). Dysregulation of the astronaut immune system in space has been identified as a major contributor to numerous spaceflight syndromes (Crucian et al., 2018). Probiotic supplements have been proposed as a countermeasure to maintain astronaut immune health during deep-space exploration (Douglas and Voorhies, 2017; Crucian et al., 2018). The DRM for a Mars mission states that all nutrients sufficient for 3 + years will be preserved and stored onboard; no resupply activity or cultivation of fresh food is planned (Anonymous, 2015). Therefore, there is a concern that the nutritional value of stored food may degrade over the course of a 3 + year Mars DRM [Human Research Roadmap (HRR) FOOD-1 section (see text footnote 2)]. It thus becomes imperative to assess the stability and shelf-life of foodstuffs, supplements, and pharmaceuticals exposed to deep space over the course of a Mars DRM. Probiotics stored at ambient temperature as freeze-dried powders in capsules are a promising option for long-term shelf-life. Most commercial probiotics are derived from the genera *Lactobacillus* and *Bifidobacterium*, although other bacterial genera (*Enterococcus*, *Streptococcus*, *Pediococcus, Leuconostoc*, *Bacillus*, and *Escherichia coli*) or fungal genera (*Saccharomyces*) are also used (Fijan, 2014). An especially suitable option for stable long-term storage are probiotics consisting of bacterial spores, which are noted both for their extreme longevity and their increased resistance to ionizing radiation (Nicholson et al., 2000; Nicholson, 2004).

Several articles in the scientific literature have advocated for the use of probiotics as a potential countermeasure for maintaining astronaut health during long-term missions into deep space (Saei and Barzegari, 2012; Urbaniak and Reid, 2016; Douglas and Voorhies, 2017; Turroni et al., 2020). However, regarding actual research reports assessing the stability and efficacy of probiotic supplementation in astronauts, the literature is sparse. One article reported on effects of simulated microgravity, supplied by a clinostat, on several phenotypic traits of a probiotic strain of *Lactobacillus acidophilus* (Shao et al., 2017). Regarding actual spaceflight, a single article has appeared in the peer-reviewed literature reporting results of a study addressing the shelf-life of a freeze-dried *Lactobacillus casei* strain Shirota probiotic, showing no changes in viability or potency after short-term (1-month) storage in the International Space Station (Sakai et al., 2018); to date no long-term studies have been reported.

We were interested in measuring the stability of freeze-dried probiotic preparations stored under ambient spacecraft conditions, including doses of ionizing radiation expected to be encountered on the spacecraft interior, during a 3-year DRM to Mars. To this end, we exposed capsules of three commercial freeze-dried probiotic bacteria (*Bifidobacterium longum, Lactobacillus acidophilus*, and spores of *Bacillus subtilis*) to simulations of Galactic Cosmic Radiation (GCR) and Solar Particle Event (SPE) radiation, supplied by the NASA Space Radiation Laboratory (NSRL).

## Materials and Methods

### Probiotic Strains and Culture Conditions

Probiotic strains used were obtained from commercial sources and are described in Table 1. *Bacillus subtilis* strain HU58 was cultivated aerobically at 37°C for 24 h on Miller LB agar plates (Miller, 1972) containing (per L): Tryptone (10 g), Yeast Extract (5 g), NaCl (5 g), and agar (15 g). Anaerobic bacteria *Bifidobacterium longum* strain BB536 and *Lactobacillus acidophilus* strain DDS-1 were cultivated on plates made from MRS broth (BD Difco) supplemented with 0.05% (final concentration) L-cysteine and agar (15 g/L). Anaerobic strains were cultivated at 37°C for 48 h in the Anaeropack system (Mitsubishi Gas Chemical America, New York, NY, United States).

**TABLE 1:** Probiotic strains used in this study.

**Strain**	**Trade name**	**Source**	**Advertised Titer (cfu/capsule)**	**Measured Titer (cfu/capsule)**
*Bifidobacterium longum* BB536	Bifido GI Balance	Life Extension, Ft. Lauderdale FL	2 × 10^9^	3.85 × 10^9^ ± 1.48 × 10^9^
*Lactobacillus acidophilus* DDS-1	Lactobacillus Acidophilus	Nutricost, Orem UT	10 × 10^9^	3.18 × 10^9^ ± 1.3 × 10^9^
*Bacillus subtilis* HU58	HU58: High-Potency *Bacillus Subtilis*	Microbiome Labs, St. Augustine, FL	5 × 10^9^	5.05 × 10^9^ ± 8.48 × 10^8^

### Viability Assays

Probiotic capsules were opened aseptically and the contents emptied into 5 mL of Peptone Water [(per L): Peptone (10 g), NaCl (5 g), pH 7.2]. This constituted the working suspension, which was vortex mixed and incubated with shaking at 37°C for 30 min. Serial tenfold dilutions of the working suspension were made in Peptone Water, plated on the appropriate medium, incubated as described above, colonies counted, and colony-forming units (cfu) per capsule back-calculated. The lower limit of detection using this assay was 100 colony forming units (cfu) per capsule.

### 16S rDNA Sequencing

Cells obtained from probiotic capsules were streak-purified on the appropriate medium and single isolated colonies were chosen for DNA isolation. Genomic DNA was purified as previously described (Cutting and Vander Horn, 1990) with the exception that *L. acidophilus* and *B. longum* cells were lysed with 100 U of mutanolysin (Sigma-Aldrich, St. Louis, MO, United States) instead of lysozyme. DNA was quantified by Qubit fluorometry (Thermo Fisher, Waltham, MA, United States). Ten nanograms of genomic DNA were amplified by PCR (OneTaq, New England Biolabs, Beverly, MA, United States) using the universal bacterial 16S primers B27F (5′-GAGTTTGATCMTGGCTCAG-3′) and B1512R (5′-AAGGAGGTGATCCANCCRCA-3′) (M = A or C; *N* = A, T, C, or G; R = A or G). PCR was performed in a PTC-200 thermal cycler (MJ Research, Waltham, MA, United States) using 35 cycles of (denaturation for 1 min at 95°C, annealing for 2 min at 55°C, and elongation for 3 min at 72°C). After a final incubation for 10 min at 72°C, the PCR products were purified using the Qiaquick PCR purification kit (Qiagen), and the purified products were sequenced at Genewiz (South Plainfield, NJ, United States). Resulting 16S rDNA sequences were searched against two online databases, the Ribosomal Database Project (RDP), Release 11.5^3^ and the National Center for Biotechnology Information (NCBI) BLASTN server^4^.

### Sample Preparation and Irradiation at Brookhaven National Laboratories/NASA Space Radiation Laboratory

**FIGURE 1:**
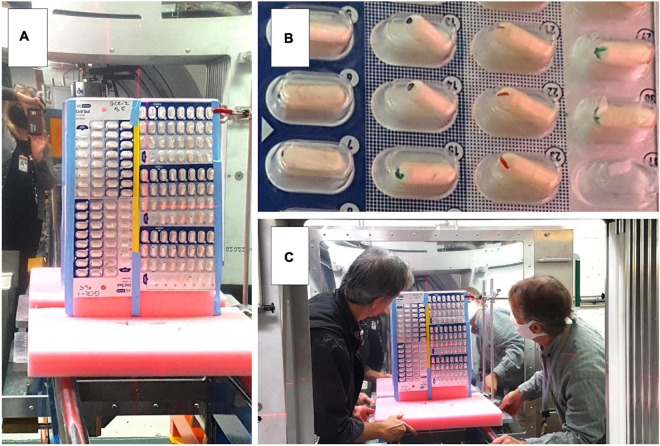
Exposure of probiotic capsules at NSRL. **(A)** Samples mounted on vertical exposure platform for GCRSim. Five 31-place blister cards were placed in the beam simultaneously. **(B)** Close-up of one blister card, showing color-coding of capsules. **(C)** Aligning samples in the beam.

Probiotic capsules were color-coded (black = *B. longum*, red = *L. acidophilus*, green = *B. subtilis*) and placed into 31- place blister cards (Apothecary Products, Burnsville, MN, United States) in random positions determined by use of an online random number generator^5^ (Figures 1A,B). Cards were shipped by commercial courier to the NASA Space Radiation Laboratory (NSRL), Brookhaven National Laboratories (BNL), Upton, NY, United States. Five cards were exposed to GCRSim and another 5 cards exposed to SPESim. Included in the package was an extra unexposed card to serve as a shipping control and a TLD dosimeter to monitor the radiation dose received during shipping. The courier was instructed to avoid exposure of the package to X-ray or e-beam scanning during shipping. The cards were mounted vertically on the exposure platform (Figure 1). The beam area was 60 cm × 60 cm and its uniformity was ± 2% across the beam area. A list of the ions and energies used is presented in Table 2. One set of samples was exposed to a total dose of 0.75 Gy of Simplified five Ion GCRSim, and a second set was exposed to a total dose of 1.0 Gy of SPESim (see Table 2 for composition of the ions used). A third set, the Shipping Control, was shipped to NSRL and back but not exposed to radiation. A dosimeter was included in the shipping package, from which it was determined to be exposed during shipping to less than 1.4 × 10^–4^ Gy of radiation, the lower detection limit of the shipping dosimeter. A fourth set of samples, the Lab Control, was stored in the laboratory for the duration of the experiment. Detailed explanations of simplified 5-ion GCRSim and SPESim can be found on the NSRL web site(^6^ and^7^), respectively.

**TABLE 2:** Ions used in simplified GCRSim and SPESim, in order of delivery.

**Ion**	**Energy (MeV)**	**Fraction of total (%)**
**1. Simplified GCRSim^1^**		

H	1,000	35
Si	600	1
He	250	18
O	350	6
Fe	600	1
H	250	39

**2. SPESim^2^**		

H	50	91.66
H	60	2.93
H	70	2.03
H	80	1.51
H	90	1.06
H	100	0.81
H	110	0.55
H	120	0.37
H	130	0.28
H	140	0.20
H	150	0.14

*^1^From https://www.bnl.gov/nsrl/userguide/SimGCRSim.php.*

*^2^From https://www.bnl.gov/nsrl/userguide/SPE-simulation.php.*

### Shelf-Life Viability of Probiotics

Probiotic capsules were loaded into blister packs, stored at ambient laboratory temperature and relative humidity (∼22–23°C and ∼40–50% R.H.) protected from light, and sampled in triplicate at intervals for viability as described above.

### Survival of Probiotics in Simulated Gastric and Intestinal Juices

Fasted State Simulated Gastric Fluid (FaSSGF) or Fasted State Simulated Intestinal Fluid (FaSSIF) (Biorelevant.com Ltd., London United Kingdom) were freshly prepared for each experiment following the manufacturer’s instructions. One probiotic capsule was emptied into 10 mL of FaSSGF or FaSSIF in a 125-mL Erlenmeyer flask and shaken at moderate speed (∼150 rpm) in a 37°C rotary shaking water bath. Aliquots were removed from FaSSGF at intervals from 0–3 h and from FaSSIF at intervals from 0–24 h, diluted serially tenfold in peptone water, plated on appropriate medium, incubated and enumerated as described above.

### Statistics

All experiments were performed on triplicate samples unless otherwise stated. Datasets were log_10_ transformed, checked for normality using the online Shapiro–Wilks calculator^8^, and tested for statistical differences by Analysis of Variance (ANOVA) using the statistical package included in the graphics program Kaleidagraph version 4.5.4 (Synergy Software).

## Results and Discussion

### Verification of Probiotic Strains

The contents of probiotic capsules from all three test organisms were cultivated on their respective solid media under either aerobic or anaerobic conditions. As expected, *L. acidophilus* and *B. longum* exhibited robust anaerobic growth and poor aerobic growth, whereas *B. subtilis* showed robust aerobic growth and poor anaerobic growth. Visual inspection revealed that all three preparations displayed homogeneous colony types, thus were presumed to be pure cultures. Phase-contrast microscopy revealed that *B. longum* and *L. acidophilus* products were phase-dark rod-shaped bacteria and that the *B. subtilis* product was phase-bright spores, as would be expected. In a previous study, several commercially available probiotic products were found to contain different bacteria than were claimed on their labels (Hoa et al., 2000). Therefore, as a quality control check we performed 16S rDNA sequencing from purified genomic DNA obtained from single isolated colonies of each probiotic strain and ran the resulting sequences through two sequence databases, the RDP and the NCBI BLASTN server. In each case, the closest match was to *L. acidophilus, B. longum*, or *B. subtilis*, respectively, thus verifying that each probiotic preparation was consistent with its labeling. Next, we determined the number of viable cells per capsule of each probiotic. In all cases, the number of viable cells per capsule corresponded to the advertised titer within a factor of three (Table 1).

### Viability of Probiotics Exposed to GCRSim and SPESim

**FIGURE 2:**
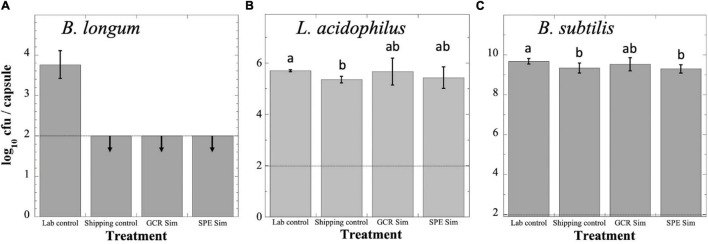
Survival of *B. longum* **(A)**, *L. acidophilus* **(B)**, and *B. subtilis* **(C)** probiotics following exposure to simulated GCR (GCR Sim) or SPE (SPE Sim) at NSRL. Capsules were exposed and processed as described in the text. Data are expressed as averages ± standard deviations (*n* = 3). Dashed line denotes lower limit of detection. Data that were not significantly different (*P* > 0.05 by ANOVA) were placed into the same group, indicated by lowercase letters. Downward arrows denote that data were below the lower detection limit of the assay (100 cfu per capsule). Note differences in magnitudes of Y-axes in each panel.

Probiotics were exposed to GCRSim and SPESim as described in section “Materials and Methods” and depicted in Figure 1, and all four sets were assayed for viability (Figure 2). Although an international standard has not to date been determined, two countries (Canada and Italy) have established the minimal therapeutic dose of probiotics as 10^9^ cfu (Hill et al., 2014). Assay of *B. longum* probiotic capsules revealed that the Lab Control had suffered a loss of > 5 logs of viability over the course of the experiment, to a titer of less than 10^4^ cfu per capsule; furthermore, the titers of the Shipping Control, GCRSim- and SPESim-exposed capsules of *B. longum* had further dropped below the level of detection (10^2^ cfu per capsule) (Figure 2A). Assay of *L. acidophilus* probiotic Lab Control capsules revealed that they had suffered a > 3-log reduction in viability over the course of the experiment, to a titer of less than 10^6^ cfu per capsule (Figure 2B). The *L. acidophilus* Shipping Control, GCRSim-, and SPESim-exposed capsules did not suffer a further decrease in viability, and their titers were not significantly different from one another (Figure 2B). Assay of the *B. subtilis* Lab Control probiotic capsules revealed no significant loss in viability over the duration of the experiment, maintaining > 10^9^ cfu per capsule (Figure 2C). In addition, the titers of the Shipping Control, GCRSim-, and SPESim-exposed *B. subtilis* capsules were not significantly different from one another (Figure 2C). In general, it appeared that exposure to GCRSim or SPESim did not significantly affect the viable titers of *L. acidophilus* or *B. subtilis* probiotics. In the case of *B. longum* probiotic, no conclusion could be reached regarding the possible effect of ionizing radiation, due to the loss of viability of the Shipping Control sample to below the detection limit during storage and shipping to and from NSRL (Figure 2A).

The results above indicating that *B. subtilis* spores and *L. acidophilus* cells were not significantly inactivated by exposure to ionizing radiation is to be expected, given the low dosages of GCRSim (0.75 Gy) and SPESim (1.0 Gy) received by samples at NSRL. A prior study showed that *L. acidophilus* survival was not significantly lowered after exposure to ionizing radiation from a ^60^Co source at doses up to 50 Gy (Gosiewski et al., 2016), and our previous experiments using wild-type spores of *B. subtilis* exposed to X-rays and heavy ions (He, Ar, Fe) revealed decimal reduction (D) values of > 300 Gy (Moeller et al., 2008). Thus, while the doses of ionizing radiation expected on a 3-year DRM to Mars might be of concern to astronauts, they are unlikely to be a significant factor in maintaining viability of freeze-dried probiotics.

### Shelf-Life of Probiotics Stored at Ambient Conditions

**FIGURE 3:**
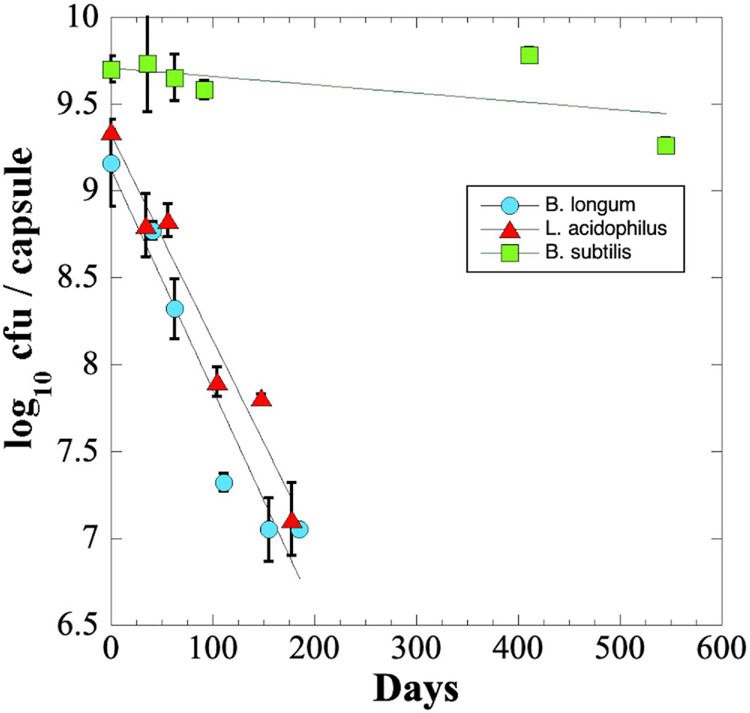
Longevity (shelf-life) of probiotic *B. longum* (blue circles), *L. acidophilus* (red triangles), and *B. subtilis* (green squares) in 31-place blister packs at ambient laboratory temperature and relative humidity. Data points are averages ± standard deviation of triplicate determinations. Best-fit lines obtained by linear regression are shown. Error bars not visible are smaller than the data points.

We were surprised that *B. longum* and *L. acidophilus* capsules in blister packs lost significant viability during their shipping to and from NSRL. To investigate this phenomenon further, we performed an experiment to measure the long-term shelf-life of probiotics stored at ambient temperatures. The results revealed that *B. longum* and *L. acidophilus* probiotic capsules lost ∼2 logs of viability in less than 200 days of storage; in contrast, *B. subtilis* spores maintained a titer of > 10^9^ cfu per capsule after 545 days of storage, the maximum time point of the experiment (Figure 3).

The rate of viability loss for each strain was further quantified from the slopes of the linear best-fit lines as the Decimal Reduction Time (*D* value) i.e., the number of days for viability to be reduced by one log_10_ (Joslyn, 1983). It was estimated that *B. longum* and *L. acidophilus* probiotic capsules exhibited *D* values of ∼77 and ∼82 days, respectively. What then could have accounted for the dramatic loss of viability observed by *B. longum* (>7 logs) and *L. acidophilus* (>3 logs) capsules shipped to and from NSRL (Figure 2)? A published study using freeze-dried powders of *B. longum* strain BB536 showed that increases in water activity (i.e., relative humidity) and temperature dramatically increased the rate of probiotic viability loss (Abe et al., 2009). Given this fact, it is plausible that excursions of relative humidity and/or temperature during storage and shipping to and from NSRL might account for the losses of *B. longum* and *L. acidophilus* viability.

The shelf-life *D* value was calculated for *B. subtilis* by extrapolation of the best-fit line (Figure 3), resulting in an estimated *D*-value of ∼1,722 days, or approximately 4.7 years. The results suggest that capsules containing *B. subtilis* spores would maintain high viability during a 3-year Mars DRM, but that *B. longum* or *L. acidophilus* probiotics would not.

### Survival of Probiotics in Simulated Gastric Fluid

**FIGURE 4:**
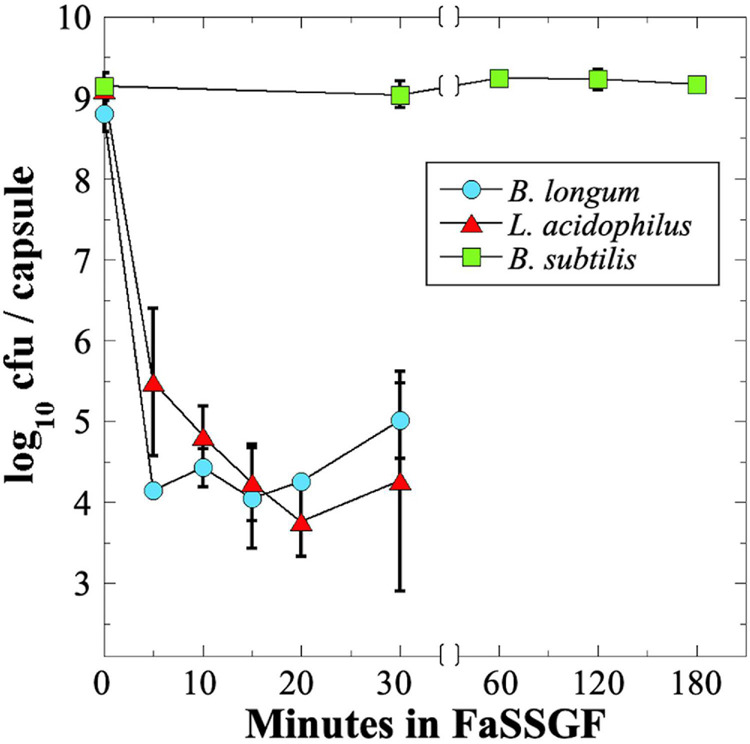
Survival of probiotic *B. longum* (blue circles), *L. acidophilus* (red triangles) and *B. subtilis* spores (green squares) in FaSSGF simulated gastric fluid. Data are averages ± standard deviations (*n* = 3). Error bars not visible are smaller than the data points. Note change in time scale after 30 min.

In order to be effective, many probiotics must survive stomach acid and bile salts to arrive at the lower GI tract in a viable state. To assess survival of probiotics through the low-pH environment of the stomach, we assayed probiotic exposure to a commercial gastric fluid simulant, Fasting-State Simulated Gastric Fluid (FaSSGF). Because of the poor survival of *B. longum* and *L. acidophilus* probiotics during storage and shipping to NSRL (Figure 2), we decided to test all three strains using fresh probiotic capsules, which all exhibited an initial titer of ∼10^9^ cfu per capsule (Table 1). We observed that exposure of the *B. longum* and *L. acidophilus* probiotics to FaSSGF resulted in a rapid decrease in viability by 4–5 orders of magnitude in the first 5 min of exposure; thereafter, their titers remained rather constant at 10^4^–10^5^ cfu per capsule until the final 30 min time point (Figure 4). We ascribe this persistent low titer to protection of a small proportion of the probiotics by association with the excipient materials present in the capsules. In contrast, the *B. subtilis* spore probiotic retained essentially 100% viability at > 10^9^ cfu per capsule after 3 h of exposure to FaSSGF (Figure 4). The results suggest that only the *B. subtilis* probiotic would be able to traverse the stomach and arrive in the small intestine at full viability.

**FIGURE 5:**
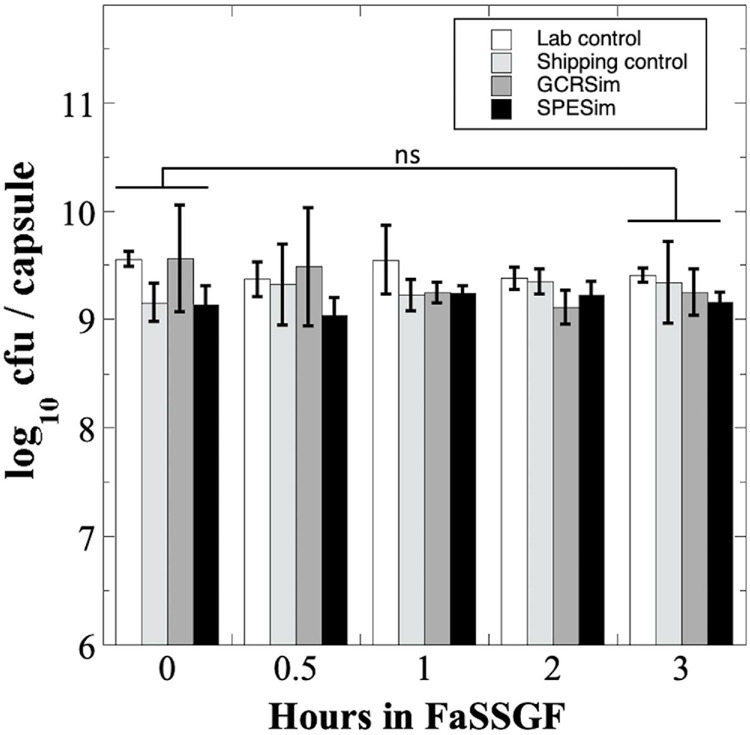
Survival of *B. subtilis* probiotics irradiated at NSRL to FaSSGF exposure. Data are averages ± standard deviation (*n* = 3). ns, not significantly different (ANOVA, *P* > 0.05).

We next tested *B. subtilis* probiotics capsules from the NSRL experiment for their survival to FaSSGF treatment (Figure 5). Exposure to FaSSGF for up to 3 h did not significantly affect the *B. subtilis* probiotic titer compared to the untreated controls for any of the Lab Control, Shipping Control GCRSim-, or SPESim-exposed probiotics (Figure 5), suggesting that ionizing radiation exposures characteristic of a 3-year DRM would not affect survival of *B. subtilis* spore probiotic during transit through the stomach.

### Survival of Probiotics in Simulated Intestinal Fluid

**FIGURE 6:**
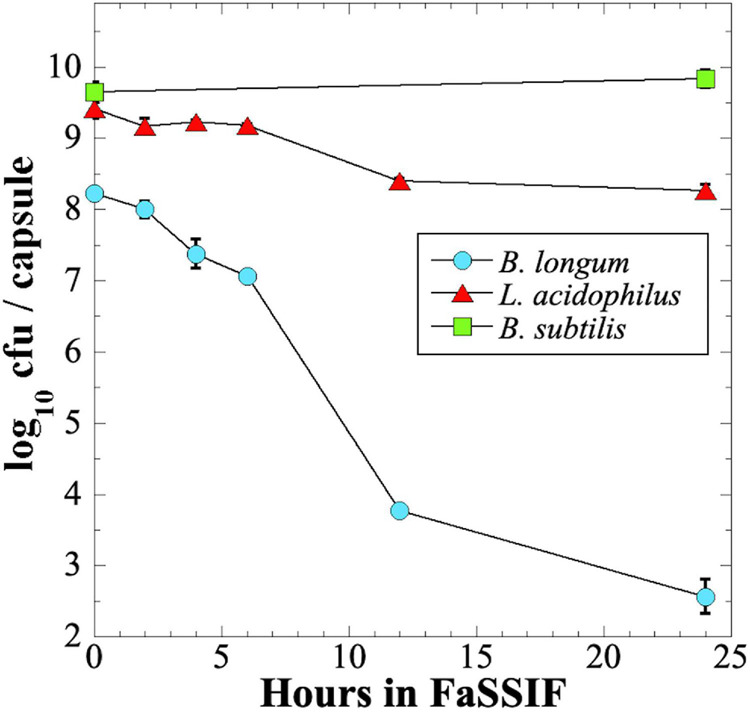
Survival of *B. longum* (blue circles), *L. acidophilus* (red triangles), and *B. subtilis* (green squares) probiotics exposed to FaSSIF. Data are averages ± standard deviation (*n* = 3). Error bars not visible are smaller than the data points.

We next tested survival of probiotic preparations when exposed over 24 h to a commercial intestinal fluid simulant, Fasting-State Simulated Intestinal Fluid (FaSSIF) (Figure 6). We observed that *B. longum* probiotic lost > 5 orders of magnitude of viability over 24 h, and > 4 logs in the first 12 h (Figure 6). In contrast, *L. acidophilus* fared much better, losing only ∼1 order of magnitude of viability in 24 h, and *B. subtilis* probiotic retained full viability after 24 h exposure to FaSSIF (Figure 6).

**FIGURE 7:**
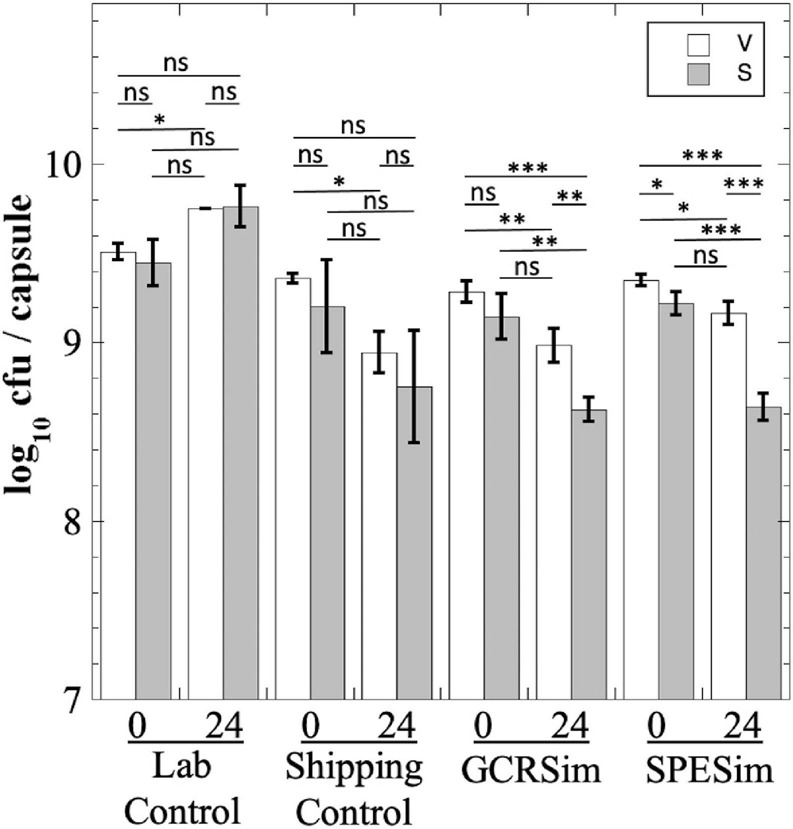
Survival of *B. subtilis* probiotic capsules from NSRL experiment exposed to FaSSIF. Total viable cells (V; open bars) and dormant, heat-resistant spores (S; gray bars) were measured. Data are averages ± standard deviation (*n* = 3). ns, not significantly different (*P* > 0.05); *P < 0.05; **P < 0.01; ***P < 0.001.

We next tested *B. subtilis* probiotics from the NSRL experiment for their survival to FaSSIF treatment; in this experiment we measured both total viable cells (V) and heat-resistant spores (S), to assess whether exposure to FaSSIF triggered spore germination (Figure 7). The Laboratory Control probiotic showed essentially no significant change in titer after 24 h in FaSSIF (Figure 7). The Shipping Control samples appeared to show a lower titer after 24 h in FaSSIF, but this was largely not statistically significant (Figure 7). In contrast, *B. subtilis* probiotic samples exposed to GCRSim and SPESim at NSRL showed small (less than 1 log) but statistically significant reductions in both total viable cells and heat-resistant spores after 24 h (Figure 7). We interpret the data as follows: exposure of GCRSim- or SPESim-treated spores to FaSSIF triggered a small proportion of spores to germinate, thus lowering S. Because FaSSIF contains no nutrients but does contain bile salts, some of the germinated spores also lost viability, leading to reduction in V. The data suggest that prior exposure to GCRSim and SPESim tended to make spores slightly more susceptible to subsequent germination in FaSSIF. Of course, the *in vitro* fasting states simulated in FaSSGF and FaSSIF do not adequately reflect the state of the GI tract in regularly fed astronauts; understanding the fate of probiotics in the gut would require *in vivo* experiments, such as have been performed in prior studies (Kailasapathy and Chin, 2000; Ranadheera et al., 2010; Bernardeau et al., 2017), and which is beyond the scope of the present communication.

## Conclusion

In evaluating the choice of probiotic(s) for inclusion on long-duration human missions outside of Earth’s protective magnetosphere, it is important to consider the ability of such preparations to maintain high viability and potency in the face of (i) long-term storage under ambient conditions and (ii) chronic exposure to low-dose radiation from solar and galactic sources. As a first attempt to address this issue, in this communication we measured the survival of three commercial probiotics to conditions expected to be encountered during a 3-year DRM to and from Mars. In all aspects tested, freeze-dried spores of *B. subtilis* maintained high viability when compared to probiotics consisting of freeze-dried *B. longum* or *L. acidophilus* cells. This finding does not mean that classic probiotic organisms should be excluded from consideration. Indeed, recent studies have found that even deliberately inactivated microbes can exert a beneficial health effect when ingested, likely due to the action of cell components or metabolites. A new definition has been established for such preparations: postbiotics (Aguilar-Toalá et al., 2018; Cunningham et al., 2021; Salminen et al., 2021).

While a long and robust shelf-life is important, a host of other factors should also be considered in choosing a probiotic for a putative Mars mission. For example, how will long-duration transit through interplanetary space affect the GI microbiome and astronaut health? What probiotic species, or mixture of species, would optimally maintain or restore healthy GI function? How will candidate probiotics perform under realistic conditions, in actual astronauts fed a diet characteristic of a Mars DRM over a 3-year period? Addressing issues such as these must be given priority if we are to deliver an optimally performing crew to Mars and return them home in good health.

## Data Availability Statement

The raw data supporting the conclusions of this article will be made available by the authors, without undue reservation.

## Author Contributions

PF-C and WN conceived the study, performed the experiments, and wrote the manuscript. Both authors contributed to the article and approved the submitted version.

## Conflict of Interest

The authors declare that the research was conducted in the absence of any commercial or financial relationships that could be construed as a potential conflict of interest.

## Publisher’s Note

All claims expressed in this article are solely those of the authors and do not necessarily represent those of their affiliated organizations, or those of the publisher, the editors and the reviewers. Any product that may be evaluated in this article, or claim that may be made by its manufacturer, is not guaranteed or endorsed by the publisher.

## Footnotes

1
https://www.nasa.gov/hrp

2
https://humanresearchroadmap.nasa.gov/

3
http://rdp.cme.msu.edu/

4
https://www.ncbi.nlm.nih.gov/

5
https://www.randomlists.com/random-numbers

6
https://www.bnl.gov/nsrl/userguide/SimGCRSim.php

7
https://www.bnl.gov/nsrl/userguide/SPE-simulation.php

8
https://www.statskingdom.com/320ShapiroWilk.html
